# Lifetime benefits of musical training

**DOI:** 10.3389/fnins.2014.00089

**Published:** 2014-04-29

**Authors:** Sébastien Paquette, Geneviève Mignault Goulet

**Affiliations:** International Laboratory for Brain Music and Sound Research, Center for Research on Brain, Language and Music, Department of Psychology, University of MontrealMontreal, QC, Canada

**Keywords:** musical training, brain plasticity, early training, transfer effect, public health

As we get older, both our bodies and brains find themselves in a constant state of change. While some of these changes are governed by normal developmental and maturational processes, others are experience-dependant and occur as a result of our day-to-day activities. Musical training is one of those activities that children tend to undertake and sometimes give up later in life. Even if of a short duration, research shows that such training may improve cognitive functioning.

Music production is a highly complex task that requires the human brain to strongly link perception and action. Indeed, when someone learns how to play a musical instrument, he/she has to develop the precise fine motor skills needed to produce the correct sounds, therefore creating a strong linkage between sensory and motor mechanisms in the brain (Zatorre et al., 2007). But little is known as to whether there are any long term benefits to such training in cases where it is discontinued early in life.

Interestingly, White-Schwoch et al. (2013), attempted to address this question via an experiment designed to determine if musical training early in life, even if for only a short period, can have long-term effects and offset the normally occurring age related decline of auditory neural function; when compared to normal hearing youg adults, older adults have a loss of temporal precision in the subcortical encoding of sound (Anderson et al., 2012). Based on the premise that adults with lifelong musical training (Parbery-Clark et al., 2012) do not exhibit age-related subcortical neural timing delays in response to fast-changing sounds (i.e., consonant–vowel (CV) transitions) important for language-based abilities, they sought to explore if limited early musical training could offset these age related timing delays.

They first divided their participants (ages 55–76) into three groups based on the level of formal music training received: *none*, *little* (1–3 years; school courses), and *moderate* (4–14 years), all of which occurred before the age of 25. The task required that participants listen to presentations of the synthesized speech syllable [da], while their *Auditory Brainstem Responses* to complex sounds (cABRs; Skoe and Kraus, 2010) were recorded. The syllable was presented in two conditions, a quiet condition (presented alone) and a masked condition (presented with a babble track).

Results show that older adults with *moderate* training had the fastest neural timing in response to the [da] stimuli followed by the *little* and *none* groups, in both conditions (quiet and masked). The *moderate* group was also the most resistant to latency delays due to noise (masked condition). Group differences were only seen in the region (time frame) of the response corresponding to the Consonant-Vowel transition (between the stop burst /d/ and the vowel /a/; the fast-changing dynamic speech elements in a syllable); during the stabilized vowel portion of the response, the groups were equivalent. The take home message here is that musical training can, to some extent, counteract age-related auditory declines even when it has been discontinued for several decades.

One cannot entirely exclude the possibility that the above-highlighted differences reflect pre-existing differences in the brain of the people that chose to study music. However, Schlaug et al. (2005) conducted a study designed to address this specific question. They compared children who were just beginning music lessons with children who did not take part in such training. After only 14 months of lessons, functional changes were observed in the temporal lobe and temporal-parietal junction. None of these differences were present between the two groups prior to the music lessons. Furthermore, Hyde et al. (2009), demonstrated structural brain changes in motor and auditory areas after only 15 months of musical training in early childhood. This is particularly interesting in the context of White-Schwoch et al. (2013)'s findings that early training, even if limited, is associated with a more efficient auditory function later in life. In Hyde et al. (2009)'s study, the structural changes were correlated with behavioral improvements in musically relevant motor and auditory skills (motor sequencing, melodic and rhythmic tests), which demonstrates the strong impact musical training can have in early childhood. Moreover, musical training in children has been shown to facilitate pitch processing not only in a musical context, but also in the context of spoken language (Magne et al., 2006). More recently, Moreno et al. (2011), showed improved vocabulary knowledge and executive function in children after only 20 days of computerized musical training, suggesting that transfer effects on cognitive skills can occur over a very short period of time. It remains to be determined whether such effects are maintained over time, or whether such a computerized musical training produces similar changes as the ones provided by a real musical training.

Similarly to what was found with children, continued music training is also related with improved cognitive functioning in older adults (Hanna-Pladdy and MacKay, 2011; Amer et al., 2013). It naturally follows that the brain of adult musicians (who had extensive training) would show significant structural adaptations that differentiate them from those of non-musicians. Interestingly, it appears that the magnitude of these changes in the brain is directly related to the amount of training received and that it is more pronounced in professional musicians than in amateur musicians (Gaser and Schlaug, 2003). This effect was also observed, although on a much shorter time scale by White-Schwoch et al. (2013), who noted a linear relationship between years of training and neural timing; more years of training were linked to faster neural responses to speech. These marked changes in the brain, both at the structural and functional level are perhaps not so surprising since musical training requires individuals to learn to pay attention to several features of sounds, such as: pitch, timing and timbre (Kraus and Chandrasekaran, 2010).

In this sense, the study by White-Schwoch et al. (2013), not only corroborates previous findings regarding the beneficial effects of musical training on the brain, but also suggests the idea that early musical training, even if short, still has an significant impact later in life. It reiterates the importance of including musical classes as an element of educational programs in public schools and provides compelling evidence supporting the idea of implementing musical training programs for underprivileged children or children with neurological disorders in order to help them reach their full potential. Additionally, music training for the elderly can be seen as a tool to delay or even attenuate age-related perceptual and cognitive declines (Alain et al., 2014) and improve subjective well-being (Seinfeld et al., 2013). Particularly, in the context of an aging population facing an increasing amount of dementia and mild cognitive impairment diagnoses, it is crucial that we attempt to maximize our cognitive potential and brain health throughout the lifespan.

## Conflict of interest statement

The authors declare that the research was conducted in the absence of any commercial or financial relationships that could be construed as a potential conflict of interest.
